# A comparison of relative survival and cause‐specific survival methods to measure net survival in cancer populations

**DOI:** 10.1002/cam4.1706

**Published:** 2018-08-01

**Authors:** Nupur Makkar, Quinn T. Ostrom, Carol Kruchko, Jill S. Barnholtz‐Sloan

**Affiliations:** ^1^ Case Western Reserve University School of Medicine Cleveland Ohio; ^2^ Case Comprehensive Cancer Center Cleveland Ohio; ^3^ Central Brain Tumor Registry of the United States Hinsdale Illinois

**Keywords:** cancer registries, cause of death, life tables, statistical data interpretation, survival analysis

## Abstract

**Background:**

Accurate cancer survival statistics are necessary for describing population‐level survival patterns and measuring advancements in cancer care. Net cancer survival is measured using two methods: cause‐specific survival (CSS) and relative survival (RS). Both are valid methodologies for estimating net survival and are used widely in medical research. In these analyses, we compare CSS to RS at selected cancer sites.

**Methods:**

Using data from 18 SEER registries between 2000 and 2014, five‐year RS and CSS estimates were generated overall as well as by age groups and by sex. To assess how closely the two survival methods corresponded, net survival percent difference was calculated with the following formula: ((RS‐CSS)/RS)*100.

**Results:**

Discrepancies between estimates obtained from CSS and RS methods varied with cancer site and age, but not by sex. In most cases, CSS was greater than RS, but cancers with available early screening and high survival rate had higher RS than CSS. Net survival percent differences were small in children and adolescents and young adults, and large in adults over the age of 40.

**Conclusions:**

While both CSS and RS aim to quantify net survival, the estimates tend to differ due to the biases present in both methodologies. Error when estimating CSS most frequently stems from misclassification of cause of death, whereas RS is subject to error when no suitable life tables are available. Appropriate use of CSS and RS requires a detailed understanding of the characteristics of the disease that may lead to differences in the estimates generated by these methods.

## 1 INTRODUCTION

Cancer survival statistics are valuable tools for researchers, physicians, and patients. Accurate estimates of survival patterns in cancer are crucial to assess the value of advancements in the field of cancer care and to estimate cancer prognosis. Cancer survival statistics can be reported in multiple forms. As net cancer survival isolates the effects of a cancer diagnosis on survival, it is a valuable statistic to describe cancer prognosis. Net cancer survival describes the probability of surviving a cancer diagnosis in the absence of competing causes of death. Net cancer survival is most frequently quantified using the following two methods: relative survival (RS) and cause‐specific survival (CSS).^1^

The majority of groups that report cancer survival statistics calculate these statistics using RS, including the National Cancer Institute's Surveillance, Epidemiology and End Results (SEER) program, the National Program of Cancer Registries United States Cancer Statistics, and the North American Association of Central Center Registries. RS estimates percent of persons surviving using all deaths adjusted for expected deaths based on life tables. RS calculations utilize life tables that estimate life expectancies for the US populations based on current age. The most likely source of error when estimating RS comes from limitations of life tables. Expected survival from life tables is not always an accurate reflection of the expected survival of a population of patients with a cancer diagnosis. Life tables cannot be generalized to all populations, and use of unsuitable life tables will lead to error when calculating RS.^2^

When life tables for a suitable reference population are not available, CSS can be used to estimate net cancer survival instead. CSS estimates percent of persons surviving using individual cause of death information. Like RS, CSS aims to estimate net cancer survival, yet the differences in methodologies lead to different measurements. While in some cases the discrepancy between the estimates from the two differing methods may be considered negligible, there are many instances in which this discrepancy may be substantial. In this study, we examine how factors such as site of cancer, sex, and age affect the correlation between the two estimates of net survival.

## 2 METHODS

This study was approved by the University Hospitals Cleveland Medical Center Institutional Review Board. Using data from 18 SEER registries between 2000 and 2014,^3^ estimates of five‐year relative survival and cause‐specific survival were calculated using the actuarial method. The actuarial method assumes that of all the cases lost to follow up, only half were at risk at the time of death. This method also makes the assumption cases are lost to follow up randomly. SEER*Stat 8.3.4^4^ was used to estimate cause‐specific survival (CSS) and relative survival (RS) at the following sites: lung and bronchus, brain and other nervous system, breast, prostate, melanoma of the skin, and acute myeloid leukemia. The justification behind selecting these sites was to include common cancers and cancers affecting multiple age groups. The sites selected also allowed us to analyze cancers with diverse characteristics (benign and malignant cancers, solid and liquid tumors, hormonal cancers, and cancers with a strong genetic component as well as cancers with a strong link to lifestyle). Estimates generated at these sites were compared overall as well as by age, sex, and behavior (for brain and other nervous system tumors only). Sites were defined using the SEER Site Recode ICD‐0‐3/ World Health Organization (WHO) 2008 recode. Age subgroups were also stratified by stage using the American Joint Committee on Cancer (AJCC) stage 6th edition 2004+ variable. The stage variable was only available for a few of the sites starting from the year 2004, so CSS and RS estimates were only compared by stage at lung and bronchus and breast from 2004 to 2014.

RS is calculated as overall observed survival for patients with a given cancer diagnosis divided by expected survival of a similar population of patients without the cancer diagnosis. When calculating expected survival, the Ederer II method was used. With the Ederer II method, matched individuals are considered at risk until the corresponding patient with a cancer diagnosis is censored or dies. When estimating RS, the default survival table was selected on SEER*Stat. The expected survival in this table comes from the US annual life tables from the National Center for Health Statistics and is based on life tables from 1970 to 2012 where individuals are matched with the appropriate estimation for age and year of diagnosis.^5^ These life tables include sex‐ and race‐specific estimates of life expectancy. The NCHS constructs these life tables using vital statistics and census data, as well as data from Medicare for ages 66‐99 years to calculate death rates. For life tables from 2000 to 2007, mortality rates were smoothed beginning at age 66. For life tables 2008 and later mortality rate were smoothed around the age of 85, but age at which smoothing began varied with race. Methodology used to generate these life tables is continuously refined and varies slightly by year.^5^

CSS is calculated as number of persons with a cancer diagnosis still living after the cancer diagnosis of interest divided by total number of persons with the cancer diagnosis of interest. Individuals who die from competing causes of death are censored from the population. At all sites, except nonmalignant brain and other nervous system, CSS estimates were generated using the SEER cause‐specific death classification.^6^ For patients with only one cancer, the SEER cause‐specific death classification attributes the following causes of death as cancer‐specific: cancer of the same site, cancer of same organ system, all malignant cancers, and site‐specific noncancer disease. At certain cancer sites, deaths coded as HIV related were also classified as cancer‐specific. For nonmalignant brain and other nervous system tumors, CSS was calculated by categorizing deaths due to in situ, benign, or unknown behavior neoplasms as cancer‐specific.

Selection criteria were adjusted to include only individuals with one cancer and individuals of known age. Cases in which cancer was reported only through a death certificate or autopsy were excluded when calculating survival. Cases with any values (including age, race, etc.) not found in expected survival life tables were also excluded. With these selection criteria, 2.13% (range: 0.83%‐2.66%) and 2.78% (range: 1.24%‐3.18%) of cases were excluded when estimating RS and CSS respectively.

To assess how closely the two survival methods corresponded, the percent difference between the two net survival estimates was calculated with the following formula:

$$\frac{\text{RS}-\text{CSS}}{\text{RS}}\times 100\%.$$

RS was used as the referent in this formula as it is more commonly used for cancer statistics reporting.

## 3 RESULTS

### 3.1 Net survival estimates by cancer sites

**Figure 1:**
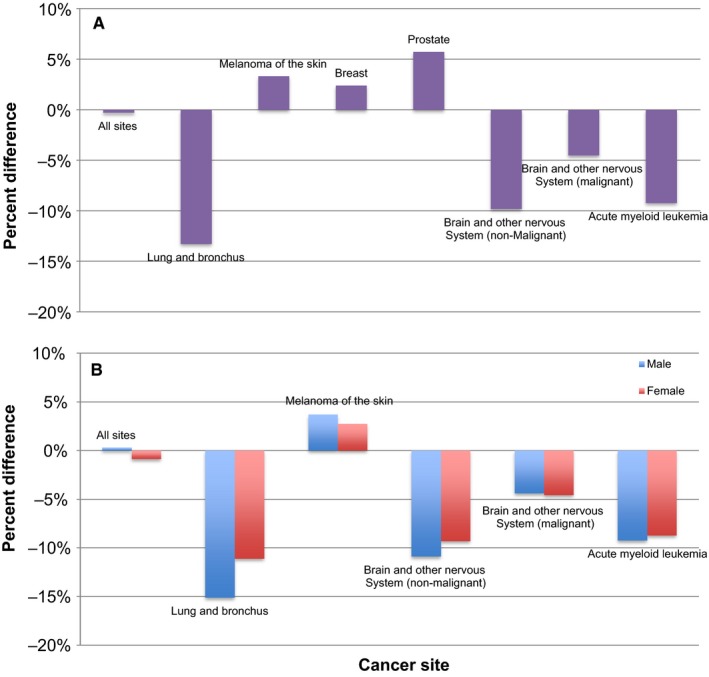
Percent differences between RS and CSS are shown at selected cancer sites (A) overall and for (B) males and females. Percent differences are based on SEER data from 2000 to 2014

Estimates for CSS and RS at all sites of cancer combined were closely related with a percent difference of only −0.3% and net difference of only −0.2%. The differences between CSS and RS estimates were greater when the cancers were separated by site (Figure 1A, Table 1). Both the magnitude and direction of percent difference between the two estimates varied with cancer site. A negative percent difference indicated that CSS estimates were greater than RS estimates and vice versa. Percent difference was negative for most cancer sites including lung and bronchus (−13.29%), brain and other nervous system (malignant: −4.52%; nonmalignant −9.83%), and acute myeloid leukemia (−9.24%). For melanoma of the skin (3.33%), breast cancer (2.40%), and prostate cancer (5.74%), RS estimates were greater than CSS estimates.

**Table 1:** 5‐Year net survival estimates, 95% confidence intervals (95% CI) and percent difference between cause‐specific survival (CSS) and relative survival (RS) at selected cancer sites (SEER 2000‐2014)

Site of cancer	N (RS)	5‐y RS, % (95% CI)	N (CSS)	5‐y CSS, % (95% CI)	Percent difference (%)	Net difference (%)
All sites	4 785 411	67.6 (67.6‐67.7)	4 753 977	67.8 (67.8‐67.9)	−0.30	−0.20
Lung and bronchus	542 517	14.3 (14.2‐14.5)	538 003	16.2 (16.0‐16.3)	−13.29	−1.90
Melanoma of the skin	284 158	96.2 (96.0‐96.3)	283 219	93.0 (92.9‐93.1)	3.33	3.20
Breast	769 803	91.8 (91.7‐91.9)	766 607	89.6 (89.5‐89.7)	2.40	2.20
Prostate	678 490	99.3 (99.2‐99.3)	674 358	93.6 (93.5‐93.6)	5.74	5.70
Brain and other nervous system	171 249	65.3 (65.0‐65.6)	170 142	71.9 (71.7‐72.2)	−10.11	−6.60
Brain and other nervous system (non‐malignant)	102 576	88.5 (88.2‐88.8)	102 177	97.2 (97.1,97.4)	−9.83	−8.70
Brain and other nervous system (malignant)	69 496	33.2 (32.8‐33.6)	68 782	34.7 (34.3‐35.1)	−4.52	−1.50
Acute myeloid leukemia	33 925	23.8 (23.3‐24.4)	33 644	26.0 (25.5‐26.5)	−9.24	−2.20

### 3.2 Net survival estimates by sex

To account for possible effects of sex on discrepancies between estimates from the two methods, results were also stratified by sex (Figure 1B, Table 2). At all cancer sites, net survival estimates were greater in females than in males. No strong pattern was noted between percent difference and sex. The percent differences between the estimates obtained from CSS and RS methods at all selected cancer sites were fairly consistent for both sexes.

**Table 2:** 5‐Year net survival estimates, 95% confidence intervals (95% CI), and percent difference between cause‐specific survival (CSS) and relative survival (RS) by selected sites and sex (SEER 2000‐2014)

Sex	Site of cancer	N (RS)	5‐y RS, % (95% CI)	N (CSS)	5‐y CSS, % (95% CI)	Percent difference (%)	Net difference
Male	All sites	2 357 142	66.3 (66.2‐66.4)	2 339 470	66.1 (66.0‐66.2)	0.30	0.20
	Lung and bronchus	290 788	11.9 (11.8‐12.1)	288 079	13.7 (13.5‐13.8)	−15.13	−1.80
	Melanoma of the skin	153 976	94.7 (94.4‐94.9)	153 379	91.2 (91.0‐91.3)	3.70	3.50
	Brain and other nervous system (non‐malignant)	33 830	87.1 (86.5‐87.6)	33 694	96.6 (96.4‐96.8)	−10.91	−12.90
	Brain and other nervous system (malignant)	38 703	31.9 (31.4‐32.4)	38 291	33.3 (32.8‐33.8)	−4.39	−1.40
	Acute myeloid leukemia	18 248	22.7 (22.0‐23.4)	18 085	24.8 (24.1‐25.5)	−9.25	−2.10
Female	All sites	2 428 269	68.9 (68.8‐69.0)	2 414 507	69.5 (69.4‐69.5)	−0.87	−0.60
	Lung and bronchus	251 729	17.1 (17.0‐17.3)	249 924	19.0 (18.8‐19.1)	−11.11	−1.90
	Melanoma of the skin	130 182	97.8 (97.6‐98.0)	129 840	95.1 (95.0‐95.3)	2.76	2.70
	Brain and other nervous system (non‐malignant)	68 746	89.2 (88.8‐89.6)	68 483	97.5 (97.4‐97.7)	−9.30	−10.80
	Brain and other nervous system (malignant)	30 793	34.9 (34.3‐35.5)	30 491	36.5 (35.9‐37.1)	−4.58	−1.60
	Acute myeloid leukemia	15 677	25.2 (24.5‐26.0)	15 559	27.4 (26.6‐28.2)	−8.73	−2.20

### 3.3 Net survival estimates by age groups

**Figure 2:**
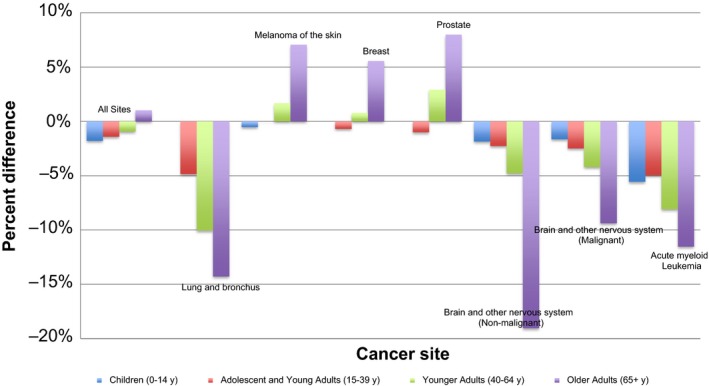
Percent differences between RS and CSS at selected cancer sites in children (0‐14 years), adolescents and young adults (15‐39 years), younger adults (40‐64 years), and older adults (65+ years) shown based on SEER data from 2000 to 2014. Due to small sample sizes, in children percent differences were not calculated for cancers at following sites: lung and bronchus, breast, and prostate

CSS and RS were estimated for children (age 0‐14 years), adolescents and young adults (AYA) (age 15‐39 years) and adults (age 40+ years [Figure 2, Table 3]). Adults were divided into two groups: younger adults (40‐64 years) and older adults (65+ years). Due to small sample size, CSS and RS were not calculated in children for cancer at the following sites: lung and bronchus, breast, and prostate. In children and AYA, the differences between the estimates obtained from the two methods were quite small (percent differences less than 6% and net differences less than 4%) at the selected sites. Discrepancies between estimates obtained from the two methods were largest in older adult cancers. When compared overall, RS was greater than CSS for melanoma of the skin, breast cancer, and prostate cancer, but when stratified by age this was only true in adults (aged older than 40 years).

**Table 3:** 5‐Year net survival estimates, 95% confidence intervals (95% CI), and percent differences between cause‐specific survival (CSS) and relative survival (RS) by selected sites and age cohorts (SEER 2000‐2014)

Age group	Site of cancer	N (RS)	5‐y RS, % (95% CI)	N (CSS)	5‐y CSS, % (95% CI)	Percent difference (%)	Net difference (%)
Children (0‐14 y)	All sites	44 037	83.1 (82.7‐83.4)	43 805	84.6 (84.2‐85.0)	−1.81	−1.50
	Melanoma of the skin	596	96.4 (94.3‐97.7)	595	96.9 (95.0‐98.1)	−0.52	−0.50
	Brain and other nervous system (non‐malignant)	2260	96.7 (95.7‐97.4)	2254	98.5 (97.8‐99.0)	−1.86	−1.80
	Brain and other nervous system (malignant)	8503	72.6 (71.5‐73.6)	8443	73.8 (72.8‐74.8)	−1.65	−1.20
	Acute myeloid leukemia	1739	64.6 (62.1‐67.0)	1726	68.2 (65.7‐70.5)	−5.57	−3.60
Adolescents and young adults (15‐39 y)	All sites	317 260	84.8 (84.7‐84.9)	315 778	86.0 (85.8‐86.1)	−1.42	−1.20
	Lung and bronchus	4388	37.1 (35.5‐38.6)	4330	38.9 (37.3‐40.5)	−4.85	−1.80
	Melanoma of the skin	37 825	96.4 (96.2‐96.7)	37 773	96.4 (96.2‐96.6)	0.00	0.00
	Breast	42 021	86.2 (85.9‐86.6)	41 820	86.8 (86.4‐87.1)	−0.70	−0.60
	Prostate	440	89.0 (85.1‐91.9)	439	89.9 (86.3‐92.5)	−1.01	−0.90
	Brain and other nervous system (non‐malignant)	10 974	97.0 (96.6‐97.4)	10 961	99.2 (99.0‐99.4)	−2.27	−2.20
	Brain and other nervous system (malignant)	11 895	68.1 (67.2‐69.1)	11 757	69.8 (68.9‐70.7)	−2.50	−1.70
	Acute myeloid leukemia	4621	54.5 (52.9‐56.0)	4588	57.2 (55.6‐58.8)	−4.95	−2.70
Younger adults (40‐64 y)	All sites	2 130 931	73.7 (73.6‐73.7)	2 119 762	74.4 (74.3‐74.5)	−0.95	−0.70
	Lung and bronchus	188 201	16.9 (16.7‐17.1)	186 594	18.6 (18.4‐18.8)	−10.06	−1.70
	Melanoma of the skin	140 987	95.8 (95.7‐96.0)	140 684	94.2 (94.1‐94.3)	1.67	1.60
	Breast	447 188	92.3 (92.2‐92.4)	445 908	91.6 (91.5‐91.7)	0.76	0.70
	Prostate	293 350	99.6 (99.5‐99.7)	292 341	96.7 (96.6‐96.7)	2.91	2.90
	Brain and other nervous system (non‐malignant)	44 753	94.2 (93.9‐94.5)	44 638	98.7 (98.6‐98.8)	−4.78	−4.50
	Brain and other nervous system (malignant)	27 468	26.2 (25.6‐26.8)	27 198	27.3 (26.7‐27.9)	−4.20	−1.10
	Acute myeloid leukemia	10 803	32.1 (31.1‐33.1)	10 711	34.7 (33.7‐35.7)	−8.10	−2.60
Older adults (65+ y)	All sites	2 294 836	59.1 (59.0‐59.2)	2 276 281	58.5 (58.4‐58.5)	1.02	0.60
	Lung and bronchus	349 832	12.6 (12.4‐12.7)	346 983	14.4 (14.3‐14.5)	−14.29	−1.80
	Melanoma of the skin	104 750	96.5 (96.1‐96.9)	104 167	89.7 (89.5‐90.0)	7.05	6.80
	Breast	280 583	91.9 (91.7‐92.2)	278 868	86.8 (86.7‐87.0)	5.55	5.10
Older adults (65+ y)	Prostate	384 663	99.0 (98.9‐99.1)	381 541	91.1 (91.0‐91.2)	7.98	7.90
	Brain and other nervous system (non‐malignant)	44 599	79.8 (79.1‐80.5)	44 334	95.0 (94.7‐95.2)	−19.05	−15.20
	Brain and other nervous system (malignant)	21 630	6.4 (6.0‐6.8)	21 384	7.0 (6.6‐7.4)	−9.37	−0.60
	Acute myeloid leukemia	16 762	5.2 (4.8‐5.6)	16 619	5.8 (5.4‐6.3)	−11.54	−0.60

**Figure 3:**
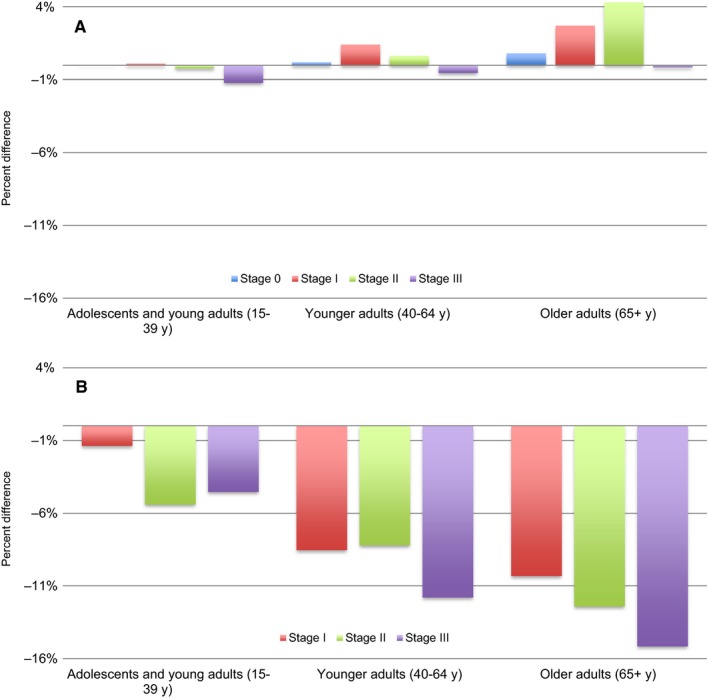
Percent differences between RS and CSS are shown stratified by age and stage for (A) cancer of breast and (B) cancer of lung and bronchus. Percent differences are based on SEER data from 2004 to 2014. Due to small sample sizes, stage 0 cancer of lung and bronchus was not included

For cancers of breast and lung and bronchus, age groups were further stratified by stage to assess for a potential confounding effect (Figure 3). Results showed that the magnitude of the discrepancy between CSS and RS estimates was related more closely to age rather than stage of cancer. Interestingly, for advanced stage breast cancer (stage III) the direction of the difference between CSS and RS was reversed (Figure 3A).

## 4 DISCUSSION

In this study, CSS and RS estimates were compared in several conditions. In many cases, the difference between CSS and RS was small indicating that in these situations CSS can be used as a reliable alternative to RS. Unlike RS estimates, CSS estimates do not rely on accurate life tables to accurately measure net survival. Measuring CSS, however, requires all causes of death to be classified as either a death attributable to cancer diagnosis or as a death not attributable to cancer diagnosis. The largest potential source of error for cause‐specific survival is misclassification of cause of death. Misclassification can be divided into two groups: genuine or conceptual.^7^, ^8^ Genuine misclassification is a problem with data collection that leads to inaccurate information on death certificates. On the other hand, conceptual misclassification is a problem that occurs when the cause of death cannot be easily categorized as attributable or not attributable to cancer diagnosis. For example, physicians may differ in how they categorize a death due to infection in a patient whose immune system has been suppressed by cancer treatment.

Misclassification of cause of death can lead to under attribution or over attribution of cancer as a cause of death, but studies show that misclassification is more likely to result in underestimation of cancer mortality. Welch and Black analyzed data from 1994 to 1998 for a study which demonstrated that 41% of deaths that occur within 1 month of cancer‐directed surgery were coded as non‐cancer‐specific deaths.^8^ Another study, using data from 1961 to 1987, reported an underestimation of cancer mortality by about 18%.^9^

In the analyses we performed, CSS was greater than RS for cancers of lung and bronchus, brain and other nervous system, and acute myeloid leukemia. This suggests overestimation of CSS and/or underestimation of RS. Under attribution of cancer as a cause of death likely lead to overestimation of CSS at these sites. Of note, CSS was calculated using the SEER cause‐specific death classification, which attempts to compensate for misclassification of cause of death. If CSS were calculated using only the cancer of interest, the difference between CSS and RS would have been greater. Another possibility is that RS was underestimated at these sites. This is particularly true in the case of lung cancer. Cohorts of lung cancer patients include more smokers than the general population. As a result, general life tables overestimate the lifespan of patients with lung cancer and underestimate relative survival. The life tables utilized by SEER*Stat to generate expected survival estimates are stratified by sex and race, but do not incorporate all variables that are shown to be significant correlated with life expectancy. In particular, increased income is significantly associated with increased life expectancy, as well as higher increases in life expectancy over time.^6^ County of residence and comorbidities are also strongly associated with life expectancy.^7^ These factors are not included in the standard life tables generated by the National Center for Health Statistics, and as a result may lead to errors in estimating the “true” expected survival for cancer patients.

Stratification by age revealed that the discrepancies between CSS and RS were most prominent in adults, especially adults older than 65 years. The effect of age on the difference between CSS and RS appeared to be independent of stage of cancer. The increasing disparity between CSS and RS in older populations can be explained by a greater amount of error when classifying cause of death in elderly patients. Literature suggests that physicians may be less precise when coding cause of death for individuals that have a higher probability of dying, including elderly patients.^10^ This and the greater prevalence of competing mortality risks may lead to a greater degree of death certification misclassification in older patients.

When net survival was calculated for breast cancer, prostate cancer, and melanoma of the skin, RS estimates were greater than CSS estimates. This can be partially attributed to the “healthy participant” effect. The “healthy participant” effect^11^ describes the phenomenon in which RS is overestimated when calculated for cancers diagnosed through screening. This is because populations who undergo regular screening have longer life spans than the general population. Stratification by age in this study demonstrated that at these sites RS was greater than CSS only in adults older than 40. As guidelines recommend screening for breast and prostate cancer starting after the age of 40,^12,13^ this further supports the notion that RS was overestimated due to the “healthy participant” effect. Of note, the direction of the difference between RS and CSS was reversed in stage III breast cancer. This suggests that for high stage breast cancer the bias from misclassification of cause of death on CSS is stronger than the bias of the “healthy participant” effect on RS.

Our study is not the first to examine whether CSS may be an acceptable alternative to RS. Other published studies compared CSS and RS estimates and explore factors that influence these estimates. A study by Hu et al^14^ published in Cancer 2013 assessed the utility of COD data from death certificates using the observed/expected ratio (O/E ratio) approach. Their study looked at how RS and CSS estimates and O/E ratios vary based on patients’ age, race, sex, and tumor stage for various solid tumors. A study by Howlader et al published in JNCI 2010^6^ also compared RS and CSS estimates at several cancer sites by calculating the net difference between RS and CSS estimates. This study investigated how factors including race, age, and socioeconomic status influence RS and CSS estimates. Both of these studies also used data from SEER for their analyses; however, the years of the data vary for each study (1992‐2004 for the study by Howlader et al; 1988‐1999 for the study by Hu et al; 2000‐2014 for our study). Our study also compared RS and CSS estimates; however, the discrepancy between the two estimates was quantified using percent difference rather than net difference or O/E ratio. Similar to the referenced studies, our study examined how the correlation between CSS and RS estimates varied with age, however, our study used age groups that are more clinically applicable. Furthermore, our study also stratified age groups by stage to demonstrate that the impact of age on percent difference between RS and CSS estimates is independent of stage. Despite utilizing different approaches and slightly different populations all three studies concluded that CSS may be a reliable surrogate to RS in several situations.

The analyses in this study were performed using data from 18 SEER registries. One of the greatest strengths of this study is the large sample size provided by SEER. While our results demonstrated that CSS is a reliable method to estimate net survival in many situations, it is important to acknowledge the limitations of the study. In our analyses, individuals with multiple primaries were excluded so that CSS estimates would be more reliable. Including only individuals with one primary allowed us to account for misclassification that occurs due to metastasis. When cancer metastasizes, cause of death may be inaccurately attributed to cancer at the site of metastasis rather than cancer of the primary site.^6^ For individuals with only one primary, it is reasonable to assume that deaths attributed to all malignant cancers are cancer‐specific deaths. This same assumption cannot be made in individuals with multiple primaries, making it difficult to account for cancers that have been miscoded on death certificates due to metastasis. Additionally, as individuals with multiple primaries have a greater amount of competing mortality risks, cause of death is more likely to be misclassified in these individuals. Also, CSS and RS were only estimated at selected, common sites of cancer, so our conclusions may not be generalizable to all cancer sites. In another study, it was suggested that there is likely to be a greater degree of misclassification of cause of death in death certificates for less common cancer sites.^2^ This suggests that the despite the conclusions of our study, CSS may not be a reliable estimate of net survival for rare cancers. The analyses in this study did not investigate the validity of CSS estimates in individuals with multiple primaries or individuals with cancers at less common sites.

## 5 CONCLUSION

RS estimates are usually preferred over CSS estimates in order to avoid error resulting from misclassification of cause of death. With improvements in quality of data on death certificates^8^ and algorithms designed to compensate for misclassification of cause of death,^9^, ^10^ CSS estimates are now more reliable. RS is usually the default methodology to measure net survival, but CSS estimates may be a more accurate estimate of net survival than RS in situations where appropriate life tables are not available. Furthermore, life table survival estimates may not be an accurate representation of populations that undergo regular screening or of populations with a high prevalence of risk factors (eg, smoking) associated with other diseases that may cause death. CSS estimates in these situations are more correct and should be used instead of RS estimates. CSS estimates can be used when reliable cause of death information is available. Misclassification of cause of death tends to be high in elderly patients; therefore, CSS estimates should be avoided in these populations.

CSS and RS are both widely used in medical research, but neither methodology is a perfect net survival estimate. CSS and RS statistics should be interpreted with caution keeping in mind the limitations of both methodologies. As accurate cancer survival statistics are necessary for describing population‐level survival patterns, and for measuring advancements in cancer care, it is important to be attentive to strengths and limitations of both methodologies when using CSS and RS to report net survival. Neither RS nor CSS is strong enough to be used as a gold standard. Understanding the biases of both methodologies will enable us to make more informed decisions on which approach to use to estimate net survival depending on the situation.

## CONFLICT OF INTERESTS

There are no conflict of interests to report.
